# The Role of Hybrid PET-MRI in the Diagnosis and Management of Cardiac Sarcoidosis—A Narrative Review

**DOI:** 10.3390/jcm15145563

**Published:** 2026-07-15

**Authors:** Salmane Nasri Elmi, Marina Sennaraj, Sharan J. Kapadia, Ali Malik, Sukruth Kundur, Łukasz Małek, Sanjay Sivalokanathan

**Affiliations:** 1 GKT School of Medicine, King’s College London, London WC2R 2LS, UK; salmane.nasri_elmi@kcl.ac.uk (S.N.E.);; 2 Mount Sinai Fuster Heart Hospital, Icahn School of Medicine at Mount Sinai, New York, NY 10029, USA; 3 Department of Nursing, Faculty of Rehabilitation, University of Physical Education, 00-968 Warsaw, Poland

**Keywords:** cardiac sarcoidosis, cardiac imaging, hybrid PET/MRI, ^18^F-FDG PET, late gadolinium enhancement, T2 mapping, inflammatory cardiomyopathy

## Abstract

**Background/Objectives**: Cardiac involvement in sarcoidosis necessitates meticulous management, with accurate and timely diagnosis being crucial. The hybrid positron emission tomography/magnetic resonance imaging (PET/MRI) modality is an emerging diagnostic tool for cardiac sarcoidosis, offering potential advantages over the use of independent PET and MRI techniques for both diagnosis and prognosis. **Methods**: Following the PRISMA guidelines for study selection, a comprehensive search using the terms “hybrid PET/MRI” and/or “cardiac sarcoidosis” identified a total of 10 eligible studies. **Results**: Patients were primarily middle-aged males. Overall, hybrid PET/MR enhanced diagnostic accuracy by identifying specific subgroups, detecting active disease, enabling detailed quantitative analysis, and assessing regional involvement. Additionally, it offered valuable insights that could improve phenotyping and prognostic predictions. The primary limitation was the longer duration of a single scan; however, the total scanning time was reduced compared to conducting two separate procedures. **Conclusions**: Hybrid PET/MR imaging offers several significant advantages over separate imaging modalities when diagnosing cardiac sarcoidosis, including increased diagnostic accuracy and comprehensive tissue characterization. Further comparative studies are needed to fully understand its clinical impact and potential limitations.

## 1. Introduction

Sarcoidosis is a multisystem autoimmune inflammatory disorder of unknown etiology characterized by the development of non-caseating granulomas in various organs, such as the heart and lungs [1]. Although the pathogenesis is poorly understood, T-cells are likely key mediators, with the disorder being associated with a high CD4:CD8 ratio [2]. Genetic and environmental factors may also contribute to dysregulation of the immune system [3]. Although prevalence is not fully understood, it varies considerably by region, from 1–5 per 100,000 in South Korea to 140–160 per 100,000 in Sweden [4].

Cardiac involvement is thought to occur in up to 25% of sarcoid patients [5]. Cardiac granulomatous infiltration usually affects the basal interventricular septum and left ventricular free wall in a patchy, non-ischemic distribution [6]. Myocardial involvement increases the risk of conduction disease, ventricular arrhythmias, heart failure, and sudden cardiac death even in patients without obvious systemic symptoms [7]. A large proportion of cardiac involvement is clinically silent; unrecognized disease may only become apparent at the time of a major arrhythmic event [8].

Given the heterogeneity and non-specific presentation of cardiac sarcoidosis (CS), timely and accurate diagnosis can be challenging [9]. There are several tools aimed at diagnosing cardiac sarcoidosis, including electrocardiography [ECG], echocardiography, cardiac magnetic resonance, and ^18^F-fluorodeoxyglucose positron emission tomography [FDG-PET], though endomyocardial biopsy showing non-caseating granulomas remains the gold-standard confirmatory test [9]. The sensitivity of biopsy, however, is limited by the focal nature of sarcoidosis, and the procedure carries a non-negligible risk [9,10].

**Figure 1:**
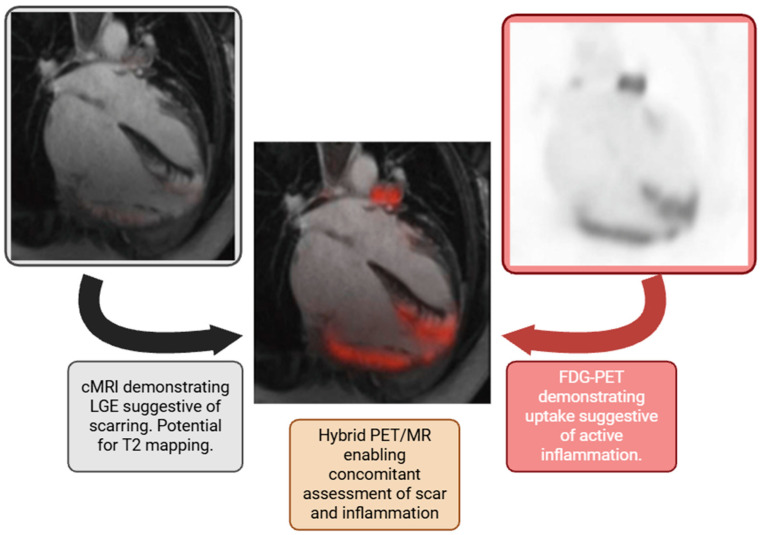
Illustration demonstrating combination of cMRI (LGE) with FDG/PET (uptake) in a single hybrid study; adapted from images from a previous case in which PET/MR distinguished the diagnosis [13].

Hybrid positron emission tomography/magnetic resonance imaging [PET/MRI] has emerged as a promising modality to address these gaps, by combining the metabolic capabilities of PET (active inflammation) with the morphological data provided by MRI (late gadolinium enhancement indicating scarring) [11] (Figure 1) PET/MRI appears in recent AHA/ACC literature [12] and has helped elucidate clinically ambiguous cases [13]; it has the potential to improve diagnostic accuracy while reducing the risks of biopsy [11]. In this review, we aim to discuss the current evidence base on hybrid PET/MRI and its use in cardiac sarcoidosis.

## 2. Methods

Study identification, screening, and selection were conducted in accordance with Preferred Reporting Items for Systematic Reviews and Meta-Analyses [PRISMA] 2020 guidelines [14]. A review protocol was developed prior to study selection. Search strategies using Medical Subject Heading [MeSH] terms were developed for all databases searched, using the key words in the title. The strategy combined MeSH and free-text terms [e.g., “Hybrid PET-MRI,” “Cardiac Sarcoidosis”] using structured Boolean operators [“AND”/“OR”]. Comprehensive searches were conducted on two databases, MEDLINE and SCOPUS, from inception to December 2025. Duplicates were removed through Rayyan (Version 1.7.0) and EndNote 2025 software [15,16].

Screening by two independent reviewers took place using Rayyan software, in line with the inclusion and exclusion criteria [15]. Any disagreements were resolved with a third reviewer. Inclusion criteria were as follows: adults [≥18 years] with suspected or confirmed cardiac sarcoidosis, with or without extracardiac sarcoidosis, as defined by study-specific or guideline-based criteria; secondly, studies in which patients underwent hybrid cardiac FDG-PET/MRI: and thirdly, studies published in peer-reviewed journals, available in full text and written in English. Exclusion criteria were unrelated studies, reviews, editorials, conference summaries, letters, individual case reports, studies focusing solely on non-cardiac sarcoidosis, and studies using PET or MRI alone without a hybrid approach. The following variables were extracted into Excel [17]: Study Characteristics (title, author, date published), Patient Demographics (sample size, age, sex), Imaging Details (set-up, PET, and CMR details), and Diagnostic Performance.

Risk of bias was independently assessed using the Joanna Briggs Institute’s (JBI) Appraisal for case series [18], a critical checklist for case series that evaluates key areas, including clarity of interventions and participant inclusion. There are 10 equally weighted domains, with a maximum score of 10.

## 3. Results

### 3.1. Study Characteristics and Imaging

**Figure 2:**
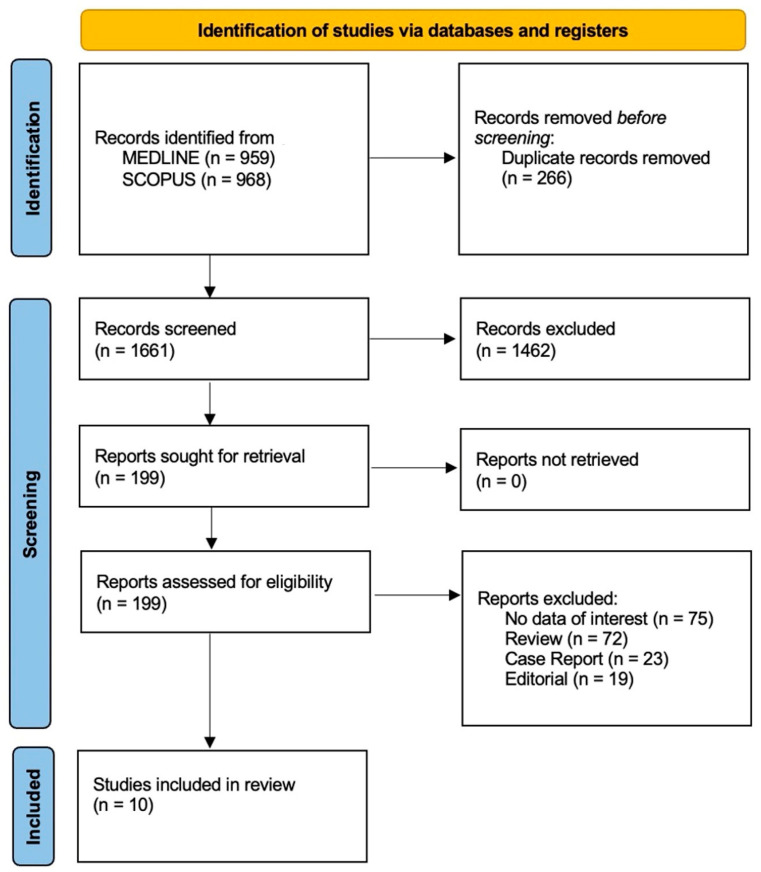
PRISMA flowchart.

A total of 1927 papers were identified through a comprehensive literature search. After applying relevant and publication filters, 199 papers remained. Subsequent full-text assessments identified 10 articles for inclusion (Figure 2). No additional articles were identified from bibliographies, and no data were from unpublished or grey literature. Reporting of funding sources across the included studies was inconsistent.

JBI risk of bias scores ranged from 8 to 9, with an average of 8.4. Mean age ranged from 48 to 63.8 years, with male predominance in most cohorts (Table 1). Pre-test probability of cardiac sarcoidosis varied considerably, with some studies enrolling patients with established disease, and others evaluating patients with suspected involvement, such as Trivieri et al. [19].

Endomyocardial biopsy-proven cardiac sarcoidosis was reported in a minority of patients, whereas extracardiac biopsy-confirmed sarcoidosis was reported in several cohorts. Follow-up duration ranged from 1.7 to 5.5 years, and prognostic analyses were performed in four studies.

All studies used ^18^F-FDG PET in combination with CMR (Table 2) Myocardial suppression protocols were broadly similar, consisting of a high-fat, low-carbohydrate dietary regimen and prolonged fasting; one study also used heparin administration. CMR assessment universally included late gadolinium enhancement. Seven studies included T2 mapping for assessment of myocardial edema, of which two analyzed T2-weighted images.

### 3.2. Diagnostic Advantages of Hybrid PET-MRI

Hybrid PET–MR studies mostly demonstrated that co-localization of focal FDG uptake with LGE improved diagnostic accuracy compared to either modality alone, and identified a distinct subgroup of patients with imaging features compatible with active cardiac sarcoidosis. In these cohorts, isolated FDG uptake without corresponding LGE was frequently attributed to inadequate myocardial suppression rather than true inflammatory involvement. Small feasibility studies comparing hybrid PET–MR with PET/CT showed similar results and confirmed the technical feasibility of simultaneous acquisition, although they were not powered for formal diagnostic accuracy assessment [20,21]. Most other studies showed some advantage. Greulich et al. showed that hybrid imaging enabled classification into active and inactive subgroups [22]. Wicks et al. [23] demonstrated that hybrid PET/MR imaging outperformed either modality alone in the detection of cardiac sarcoidosis. Similarly, Cheung et al. [24] demonstrated that the combination of FDG and LGE provided the highest overall Diagnostic Performance (AUC 0.73). Vita et al. demonstrated that combined PET and CMR findings provided greater diagnostic confidence than either modality alone in patients undergoing standard clinical imaging pathways [25]. Marschner et al. [26] showed superior Diagnostic Performance (AUC 0.84) as well as lower radiation exposure and procedure time for combined PET/MR versus standard of care separate imaging. Dweck et al. [27] showed that the target-to-normal myocardium ratio provided superior differentiation of co-localized MR+ PET+ patterns compared with SUV-based measures. Okune et al. [28] showed that hybrid PET/MR improved Diagnostic Performance compared to PET alone, and their segmental analysis using co-registered PET and CMR datasets shows that hybrid imaging enables regional assessment of inflammatory activity.

Across hybrid PET–MR studies, three principal imaging phenotypes were described: co-localized FDG uptake and LGE, representing active inflammation; LGE without FDG uptake, consistent with chronic scar; and absence of both markers, indicating no imaging evidence of cardiac involvement. This combined phenotyping enabled differentiation between active and inactive disease states within the same cohort. Wicks et al. [23] reported poor inter-method agreement [kappa = 0.021] between regional ^18^F-FDG uptake and regional LGE distribution at the time of imaging, reflecting varying disease stages across myocardial regions.

Diffuse global FDG uptake patterns were observed in control populations and in patients with inadequate myocardial suppression, whereas focal or focal-on-diffuse uptake co-localizing with LGE was strongly associated with clinically probable cardiac sarcoidosis. Interestingly, parametric mapping identified myocardial abnormalities in a subset of patients without LGE. Although T2-weighted imaging and T2 mapping may provide additional information regarding myocardial edema and active inflammation, their incremental diagnostic value beyond FDG uptake and LGE co-localization remains uncertain. Cheung et al. reported diagnostic and prognostic utility using combined PET/MRI with T1/T2 mapping, while Greulich et al. suggested that CMR mapping, particularly T1 mapping, may identify myocardial involvement in selected patients without clear LGE [22,24]. Marschner et al. also incorporated T1, T2 and extracellular volume mapping within a hybrid PET/MRI protocol, although Diagnostic Performance was primarily reported around co-localized FDG uptake and LGE patterns typical for cardiac sarcoidosis [26]. Importantly, no included study directly compared FDG/LGE-based assessment with versus without T2-based parameters. Therefore, current evidence supports T2 mapping as a potentially complementary marker rather than a substitute for FDG-based assessment, and further prospective studies are required to define its true incremental diagnostic value [22,24,26]. However, the reported utility of T2-weighted imaging and T2 mapping was inconsistent across studies, with some cohorts suggesting improved detection of active inflammation while others demonstrated limited incremental diagnostic contribution beyond conventional hybrid PET/MRI findings.

### 3.3. Prognostic Value and Monitoring

Four studies assessed the prognostic value of hybrid or combined PET–MR findings. Firstly, Trivieri et al. [19] found that hybrid PET–MR results correlated with cardiac-related outcomes during long-term follow-up, highlighting the potential of combined metabolic and structural imaging for risk stratification; the primary outcome (cardiac arrest, ventricular tachycardia, or secondary prevention implantable cardioverter-defibrillator implantation) was predicted significantly better with co-localization of LGE and FDG than either alone. Calculation of the target-to-normal myocardium ratio was also prognostically useful, with an AUC of 0.77 for separating patients with and without the primary outcome. Wicks et al. [23] similarly included follow-up to examine the link between hybrid imaging results and clinical outcomes—the primary composite endpoint (mortality, aborted sudden cardiac death, sustained ventricular arrhythmia, complete heart block, and hospitalization with decompensated heart failure) were predicted best by concomitant PET/MR findings. Similarly, Cheung et al. [24] found that co-localized LGE and FDG was the best predictor of major adverse cardiac events over a follow up of just under 2 years. Vita et al. showed that the presence of both abnormal PET and CMR findings was associated with increased risk of adverse clinical events compared with abnormalities in only one modality [25]. Hybrid imaging also influenced clinical management. In several studies, the addition of PET data to CMR reclassified disease likelihood and informed decisions regarding immunosuppressive therapy and device implantation.

### 3.4. Limitations of PET/MRI

PET/MRI may require a longer imaging time. Marschner et al. found that the imaging duration for hybrid PET/MRI [122 ± 15 min] exceeded that of PET/CT [90 ± 20 min] and MR [51 min ± 9 min] separately; however, when compared to the cumulative duration of performing both PET/CMR and CMR separately, hybrid PET/CMR unsurprisingly resulted in a significantly lower total imaging time of 43% [26]. Furthermore, PET/MRI has limited utility in patients with metallic hardware, such as implanted cardiac devices. This is especially relevant in patients with CS who may frequently require ICDs or pacemakers for disease management. Finally, there are additional practical barriers such as cost and the relatively infrequent availability of PET/MR scanners.

### 3.5. T2-Weighted Imaging and T2 Mapping

By detecting myocardial edema, T2-weighted imaging or mapping on MRI may provide additional information suggestive of myocardial inflammation, injury, or local tissue reaction. Although it does not quite demonstrate active inflammatory cell activity as FDG does, it is interesting to consider how well FDG and T2 data correlated in the reviewed studies, and whether the inclusion of T2 data could improve Diagnostic Performance. Seven studies included T2 mapping [19,21,22,24,26,27,28], of which two also analyzed T2-weighted images. In Hanneman et al. [21], although correlation between FDG and T2 was not reported, T2 was present in 50% of patients, and LGE in 66.7%, without significant correspondence, suggesting the inclusion of both LGE and T2 in the MRI component of PET/MRI may capture more patients with CS; indeed, the sensitivity of PET/MR this study was 100%, higher than in all others, although the sample was very small and specificity was not reported. In Okune et al. [28], correlation between active inflammation on T2-weighted CMR and PET was low, but sensitivity of PET/MR was good at 83.3% and specificity was the highest among all studies at 93.8%. With regard to T2 mapping, in Trivieri et al. [19], although not quite reaching statistical significance, there was a trend that MRI + focal PET showed higher T2 mapping values than MRI + negative PET (and MRI + diffuse PET), suggesting that adding T2 mapping may highlight focal active disease. In Cheung et al. [24], 46% of patients had co-localized T2 mapping and FDG, and when this was present, it had the highest specificity for CS diagnosis, even more specific than LGE + FDG. Nevertheless, in Greulich et al. [22] and Okune et al. [28], T2 mapping results did not correlate well with FDG. In both Hanneman et al. [21] and Marschner et al. [26], correlation between T2 mapping and FDG was not reported.

## 4. Discussion

The studies demonstrate that the concurrent assessment of FDG and LGE is technically feasible and has the potential to improve diagnostic accuracy and prognostication with a lower radiation burden than PET/CT (Table 3). Moreover, it may help with identifying patient subgroups, differentiating between active and chronic disease, delineating regional involvement, and enabling the calculation of additional quantitative parameters (such as the target to normal myocardial ratio). This approach also provides new insights into imaging findings; for example, some FDG/LGE mismatches have been linked to insufficient myocardial suppression. Furthermore, the ability of hybrid quantitative parameters such as the target to normal myocardial ratio to accurately distinguish true inflammatory activity could have a significant clinical impact, enhancing the precision, duration, and dosing of anti-inflammatory therapies. PET/MRI also provided insights that may enable more precise phenotyping; for example, different FGD/LGE combinations seemed to indicate varying disease stages. Using artificial intelligence to run unsupervised learning on a dataset containing paired FGD/LGE information could reveal new phenotypes. Recent prospective multimodality imaging studies in other cardiovascular settings have similarly demonstrated the incremental diagnostic value of integrated PET/MRI, particularly in cases where standalone structural imaging is inconclusive [29]. In this study, the addition of PET-derived metabolic information to MRI improved diagnostic sensitivity and lesion characterization by resolving equivocal findings and enabling more accurate distinction between active disease and chronic structural abnormalities. This supports the concept that, in cardiac sarcoidosis, hybrid PET/MRI may provide the greatest clinical value in diagnostically challenging or high-risk patients (particularly where initial investigations such as echocardiography, EKG and CMR are incongruent or inconclusive, or where these results do not fit the clinical picture), where simultaneous assessment of inflammation and fibrosis may improve diagnostic confidence and influence management decisions. However, evidence for clear superiority over sequential PET/CT and CMR remains limited, particularly in the absence of head-to-head randomized trials comparing the modalities.

Studies suggest that PET/MRI can provide valuable prognostic information, which is consistent with a previous meta-analysis [29]. MRI findings are linked to clinical outcomes, as is known [30,31], but importantly, PET/MRI shows strong correlation as well; additionally, the presence of both FDG and LGE signals indicates a poorer prognosis, which is expected since this suggests active inflammation alongside ongoing scar formation. Myocardial scarring serves as an arrhythmogenic substrate in CS [7,32,33], and detecting concurrent active inflammation may help refine patient selection for anti-inflammatory treatments or prophylactic ICD placement [34]. An important drawback in current sarcoidosis management is that currently, although serial PET imaging can be used to monitor treatment response, there is limited evidence that PET data (and indeed isolated MRI data) can be relied upon for adjustment or tapering of immunosuppression [35]. Combined PET–MRI may provide a much-needed solution to this issue, as supported by Trivieri et al. where simultaneous PET/LGE showed superiority for predicting clinical outcomes; this could enable robust image-guided titration of therapy, which is particularly useful in inflammatory diseases since excessively intense or lengthy immunosuppression would carry significant adverse effects. It may also shape timelines towards device therapy, by identifying the particularly high-risk subgroup of inflammation superimposed on scarring, leading to potentially earlier ICD implantation. Even more encouraging is the fact that PET/MRI involves less radiation exposure than PET/CT, making it a preferable option for follow-up in patients with CS [11]—in young patients, increased cumulative radiation exposure would significantly increase the risk of secondary malignancies. Direct comparison to assess whether PET/MRI is superior to standard PET as a basis for adjusting immunosuppression should be a future research priority.

The limitations of PET/MRI include increased scan duration and restricted applicability for patients with implants, and infrequent availability of hybrid scanners. However, these drawbacks are arguably outweighed by its advantages, as simultaneous imaging eliminates the need for additional tests and reduces overall time burden. Additionally, many patients who are unable to undergo PET/MRI due to implanted devices would also be unsuitable for standard MRI. It is also possible that some of the supplementary information obtained through hybrid imaging may constitute ‘noise,’ potentially leading to false-positive results. For example, there are other infiltrative or inflammatory disorders that may mimic cardiac sarcoidosis on multimodal imaging. Conditions such as Erdheim–Chester disease may present with pericardial involvement, myocardial abnormalities, and increased FDG uptake, potentially resulting in imaging appearances that overlap with cardiac sarcoidosis. Therefore, PET/MRI findings should always be interpreted within the broader clinical context and, where appropriate, alongside histological or extracardiac diagnostic data [36].

T2 mapping was of particular interest as it could theoretically substitute for FDG in complementing MRI via inflammation detection, but studies were inconclusive on its diagnostic benefit; this difference may be due to several factors, in particular the study populations included; it is plausible that in populations such as Greulich who exclusively included patients with biopsy-proven extracardiac sarcoidosis, cardiac disease may be more established or frequent, limiting the additional diagnostic utility of T2 mapping; however in studies with less well-defined populations, such as Trivieri et al. [19], where only around half of patients had biopsy proven extracardiac sarcoidosis, T2 mapping may have a greater diagnostic contribution. Crucially, there was no direct comparison available between FDG + PET with versus without T2 in terms of diagnostic accuracy, which should be a future research priority. It should also be noted that T2-weighted imaging may have drawbacks, including artefact [37,38]. Therefore, at this time, T2 mapping is likely best viewed as a complementary modality, rather than a replacement to FDG in the detection of inflammation.

### 4.1. Study Limitations

Several limitations of this review should be acknowledged. First, the literature search was restricted to MEDLINE and SCOPUS, which may have introduced indexing bias through omission of relevant studies indexed in other databases such as Embase or CENTRAL. Second, the current evidence base is limited by small and heterogeneous study populations, with substantial variation in diagnostic criteria, imaging protocols, and definitions of scan positivity across studies. Third, most included studies were single-centre prospective cohort or case-series studies with relatively limited follow-up, reducing generalisability of findings. Finally, the absence of randomized or comparative trials and the novelty of hybrid PET/MRI raise the possibility of publication bias, as studies reporting favourable findings may be more likely to be published.

The lack of a uniform standard for defining cardiac sarcoidosis across studies may lead to classification bias and is a key limitation of the evidence base [9]. This issue is especially important because clinical diagnostic criteria are often used instead of histology-based confirmation. Additionally, the criteria for defining scan positivity were inconsistent across studies, which limits the ability to directly compare the Diagnostic Performance of PET/MRI. Several studies used relatively permissive positivity criteria, whereby PET/MRI positivity could be assigned based on abnormalities identified on either imaging modality or broader hybrid imaging patterns, whereas others used a more restrictive definition, classifying a scan as positive only when there was co-localization of focal ^18^F-FDG uptake and LGE. This difference likely explains the variation in reported diagnostic specificity and sensitivity between studies, as seen from Wicks et al. versus Marschner et al. [23,26]. Specifically, more permissive criteria (e.g., Wicks et al.) increased sensitivity by capturing a broader range of potentially abnormal findings, resulting in a sensitivity of 94% but a specificity of only 44%, reflecting a higher likelihood of false-positive classification. In contrast, the more restrictive co-localization criteria (Marschner et al.) improved specificity to 96% but reduced sensitivity to 71%, as requiring simultaneous evidence of structural and metabolic abnormality may exclude patients with discordant but potentially clinically relevant findings [23,26]. The variability is also increased by the lack of standard procedures for 18-FDG suppression, which probably impacts the accuracy of myocardial 18-FDG uptake assessments across studies [39]. Moreover, there was inconsistency in MRI procedures, with some studies using T2 mapping and others only LGE detection, whereas for the best results all modalities should be included [40]. Furthermore, although literature on the usefulness of cardiac PET/MRI in CS continues to grow, most of the current evidence comes from single-centre cohort studies involving small populations.

### 4.2. Future Directions

Larger multi-centre prospective trials comparing isolated and hybrid imaging directly, utilizing standardized diagnostic criteria and imaging protocols, would yield valuable insights. Emphasis should be placed on studies directly comparing FDG/LGE-based assessment with and without T2-based parameters to determine whether multiparametric MRI provides meaningful incremental diagnostic value beyond conventional hybrid PET/MRI. Additionally, longitudinal follow-up data are essential to better understand the role of hybrid PET/MRI in therapeutic decision-making and treatment monitoring. Hybrid FDG-PET/MRI is unlikely to replace modern CMR for CS diagnosis (particularly with T2 mapping) but may add value in suspected active cardiac sarcoidosis by concomitantly detecting metabolically active inflammation especially when edema on T2-weighted imaging or T2 mapping is absent, subtle, or difficult to distinguish from chronic fibrotic injury [41,42].

## 5. Conclusions

Hybrid cardiac PET/MRI is an innovative multiparametric imaging technique offering promising diagnostic and prognostic capabilities for cardiac sarcoidosis. It allows for the concurrent assessment of myocardial metabolic activity and structural abnormalities. To confirm these initial results and facilitate the integration of PET/MRI into existing diagnostic guidelines, future large-scale, multicenter, prospective studies are necessary. These studies should feature standardized imaging protocols, diagnostic criteria, and follow-up data.

## Figures and Tables

**Table 1:** Study characteristics.

Title	Author	Type	Aim	n	Male (%)	Mean Age (y)	Age Range	Pre-Scan CS	EMB-Proven CS	Extracardiac CS	Follow Up (y)
Diagnostic accuracy and prognostic value of simultaneous hybrid 18FFluorodeoxyglucose Positron emission tomography/magnetic resonance imaging in cardiac sarcoidosis	Wicks et al.	Prospective cohort study	Diagnostic Accuracy & Prognostic Value	51	61.0	50.0	13.0	33	7	44	2.2
Same day Comparison of PET/CT and PET/MR in patients with cardiac sarcoidosis	Wisenberg et al.	Prospective case series	Compare with PET/CT	10	60.0	60.0	10.0	NA	0	5	NA
tableInitial Experience with simultaneous 18-FDG PET/MRI in the Evaluation of Cardiac Sarcoidosis and Myocarditis	Hanneman et al.	Prospective case series	Compare with PET/CT	10	40.0	56.1	9.6	7	0	7	NA
Combined simultaneous FDG-PET/MRI with T1 and T2 mapping as an imaging biomarker for the diagnosis and prognosis of suspected cardiac sarcoidosis	Cheung et al.	Prospective cohort study	Diagnostic Accuracy & Prognostic Value	42	67.0	53.0	13.0	13	0	20	1.7
Combined FDG PET/MRI versus Standard-of-Care Imaging in the Evaluation of Cardiac Sarcoidosis	Marschner et al.	Prospective cohort study	Compared with PET/CT, Cardiac MRI, and Technetium 99m sestamibi SPECT perfusion imaging	40	65.0	54.0	14.0	NA	NA	NA	NA
Hybrid Magnetic Resonance Imaging and Positron Emission Tomography with Fluorodeoxyglucose to Diagnose Active Cardiac Sarcoidosis	Dweck et al.	Prospective cohort study	Evaluate the diagnostic utility and differentiating active inflammation from inactive scar and physiological FDG uptake	25	48.0	55.0	10.0	7	NA	18	NA
Hybrid Cardiac Magnetic Resonance/Fluorodeoxyglucose Positron Emission Tomography to Differentiate Active From Chronic Cardiac Sarcoidosis	Greulich et al.	Prospective cohort study	Diagnostic utility of hybrid PET/MRI to detect and differentiate active from chronic cardiac sarcoidosis	43	65.0	48.0	11.0	43	NA	43	NA
Hybrid Magnetic Resonance Positron Emission Tomography Is Associated With Cardiac-Related Outcomes in Cardiac Sarcoidosis	Trivieri et al.	Prospective cohort study	Evaluate qualitative and quantitative assessments of hybrid MR/PET imaging in CS and to evaluate its association with cardiac-related outcomes	148	63.0	56.5	9.0	148	NA	NA	5.5
Diagnostic utility of fusion (18)F-fluorodeoxyglucose positron emission tomography/cardiac magnetic resonance imaging in cardiac sarcoidosis	Okune et al.	Retrospective cohort study	Evaluate the diagnostic value of fusion PET/CMR imaging by characterizing the inflammatory phase in each segmental region in patients with suspected CS	74	52.8	63.8	12.8	NA	NA	54	NA
Complementary Value of Cardiac Magnetic Resonance Imaging and Positron Emission Tomography/Computed Tomography in the Assessment of Cardiac Sarcoidosis	Vita et al.	Retrospective cohort study	Assess complementary diagnostic and prognostic value of hybrid PET/MRI	107	69.0	55.0	11.0	NA	NA	32	1.7

**Table 2:** FDG-PET/CMR Protocols.

Study	PET Tracer	FDG Suppression Protocol	PET Evaluation Parameters	CMR Evaluation Parameters
Wicks et al.	18FDG	24 h High fat content --> 12 h prolonged fast	Qualitative (18F-FDG uptake) Quantitative: SUV max:least avid ratio	LGE (qualitative)
Wisenberg et al.	18FDG	Fast 12 h prior scanning -->High fat, low carb diet day before scan --> UH 5 lU/kg + UH 10 lU/kg	Qualitative (18F-FDG uptake) Quantitative: SUV max	LGE (Qualitative)
Hanneman et al.	18FDG	>/= 12 h High fat, high protein, low carbohydrate diet	Qualitative (18F-FDG uptake)	LGE and T2W1 (Qualitative)
Cheung et al.	18FDG	24 h high fat, high protein, low carb diet --> complete fast 12 h before imaging	Qualitative (18F-FDG uptake) Quantitative: Cardiac Metabolic Volume (CMV) (defined using SUV threshold: myocardium above 1.2× LV blood pool: cardiac max SUV ratio, per their method)	LGE (Qualitative], Native T1 mapping (global + maximum segmental; abnormal if >1286 ms)
Marschner et al.	18FDG	high-fat, high-protein, low-carbohydrate diet for the entire day before 18F-FDG PET/CT imaging --› complete fast with the exception of water for the immediate 12-h period prior to imaging	Qualitative (18F-FDG uptake)	Any LGE or LGE in a pattern typical of CS (Qualitative); Maximum native T1, T2, and extracellular volume were defined as the highest segmental value for each parameter, and values were categorized as normal or abnormal based on established scanner specific local reference ranges (elevated T2 values > 45 s; elevated T1 values > 1289 s; and elevated extracellular volume > 30%) (Quantitative)
Dweck et al.	18FDG	High-fat, low-carbohydrate diet + 224 h carbohydrate restriction + 212 h fasting prior to scan	Qualitative: visual co-localization of FDG uptake with LGE	LGE pattern (non-coronary distribution), cine ventricular function, T2 mapping
Greulich et al.	18FDG	High-fat low-carbohydrate diet + 212 h fasting	Qualitative FDG uptake pattern ± SUV	LGE, cine ventricular function
Triviera et al.	18FDG	High-fat low-carbohydrate diet + fasting	Qualitative FDG uptake pattern	LGE, ventricular function
Okune et al.	18FDG	High-fat low-carbohydrate diet with prolonged fasting	Qualitative FDG uptake pattern ± SUV	LGE presence and distribution, ventricular function
Vita et al.	18FDG	High-fat low-carb diet + fasting	Qualitative FDG uptake pattern	LGE, ventricular function

**Table 3:** Summary table of hybrid PET/MR versus standalone PET/CT and MR.

	Hybrid PET/MR	Standalone PET/CT	Standalone MR
Diagnostic and prognostic value	Possible improved diagnostic accuracy compared to isolated imaging via perfect spatial co-registration in a patchy, focal disease. Possible prognostic advantages over isolated imaging especially for the FDG+/LGE+ phenotype, resulting in identification of a higher-risk subgroup which may benefit from earlier, simultaneous device and immunosuppressive therapy.	Characterization of inflammatory burden, demonstrating active disease.	Characterization of regions of LGE, demonstrating scarring.
Safety	Reduced ionizing radiation burden compared to standalone PET/CT.	Highest ionizing radiation burden.	No ionizing radiation.
Limitations	Increased scanning time, reduced availability of hybrid scanners.	High radiation burden, inability to characterize scar tissue.	Inability to characterize active inflammation; T2 mapping may be promising (using edema as a surrogate for inflammation) but evidence is lacking for ability to replace FDG evaluation.

## Data Availability

As this is a narrative review, there is no research data per se; further details on study selection are available from the corresponding author on reasonable request.

## Abbreviations

The following abbreviations are used in this manuscript:

PET/MRI	positron emission tomography/magnetic resonance imaging
AHA/ACC	American Heart Association/American College of Cardiology
JBI	Joanna Briggs Institute
SUV	Standardized uptake value
